# Interactive visualization of brain-scale spiking activity

**DOI:** 10.1186/1471-2202-14-S1-P110

**Published:** 2013-07-08

**Authors:** Christian Nowke, Bernd Hentschel, Torsten Kuhlen, Maximilian Schmidt, Sacha J van Albada, Jochen M Eppler, Rembrandt Bakker, Markus Diesmann

**Affiliations:** 1 Virtual Reality Group, RWTH Aachen University, Aachen, Germany; 2 Institute of Neuroscience and Medicine (INM-6) and Institute for Advanced Simulation (IAS-6), Jülich Research Centre, Jülich, Germany; 3 Donders Institute for Brain, Cognition and Behavior, Radboud University, Nijmegen, The Netherlands; 4 Medical Faculty, RWTH Aachen University, Aachen, Germany; 5 JARA - High-Performance Computing, RWTH Aachen University, Aachen, Germany

## 

In recent years, the simulation of spiking neural networks has advanced in terms of both simulation technology [1,2] and knowledge about neuroanatomy [3,4]. Due to these advances, it is now possible to run simulations at the brain scale [5,6], which produce an unprecedented amount of data to be analyzed and understood by researchers.

To aid computational neuroscientists with the development of models and especially with the visual inspection and selection of data for analysis, we developed VisNEST [7], a tool for the combined visualization of simulated spike data and anatomy. This provides a rapid overview of the relationship between structure and activity. VisNEST currently uses spike data from the neural simulation tool NEST [1] and geometry from the Scalable Brain Atlas [4], but is not limited to these tools.

In our contribution we will present VisNEST using a Picasso 3D system, which allows users to interactively investigate and explore the simulated data from a large-scale model of 32 vision-related areas of the macaque [6]. The system is equipped with infrared tracking and uses passive glasses to render the image for the user standing in front of the screen.

**Figure 1:**
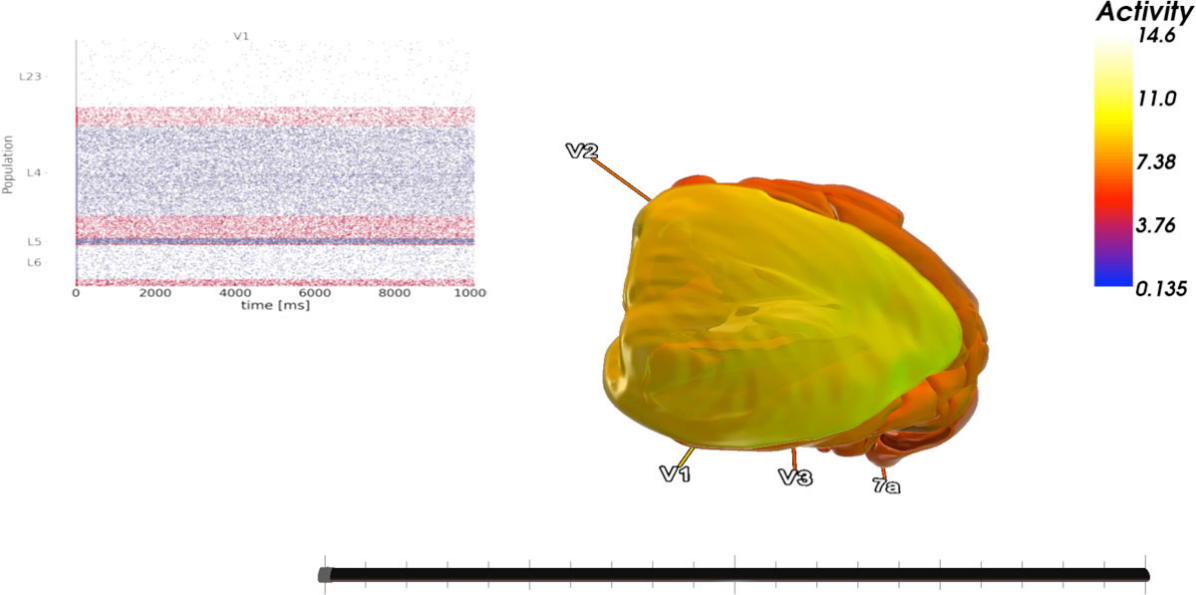
**Main view of the simulated activity data**. The mean spiking activity of the different areas is shown by color. The optional dot plot shows the spikes from the currently selected area.
